# Epididymal leiomyosarcoma: a case report

**DOI:** 10.1007/s12672-026-04443-0

**Published:** 2026-01-20

**Authors:** Qingdian Cong, Jiaying Liu, Zhilan Huang, Ruoling Gao, Huiqing Zhang, Jibo Hu

**Affiliations:** Department of Radiology, The Fourth Affiliated Hospital of School of Medicine, and International School of Medicine, International Institutes of Medicine, Zhejiang University, Yiwu, China

**Keywords:** Epididymal leiomyosarcoma, Clinical manifestations, Imaging features, Diagnosis, Treatment, Surgery

## Abstract

**Background:**

Epididymal leiomyosarcoma is a rare, malignant neoplasm (Fisher et al. in Am J Surg Pathol 25(9):1143–9, 2001) characterized by rapid growth and aggressive behavior. Over the past decade, fewer than 30 cases of epididymal leiomyosarcoma have been reported globally.

**Case presentation:**

In 2024, a male presented to the Fourth Affiliated Hospital of Zhejiang Universityl with a left scrotal mass. The patient underwent scrotal ultrasound and pelvic MRI, initially diagnosed with a hypervascular tumor of the epididymis; however, the precise nature of the tumor remained indeterminate before surgery. Following radical orchiectomy, postoperative pathology confirmed the diagnosis of epididymal leiomyosarcoma.

**Conclusion:**

Although epididymal leiomyosarcoma represents a rare entity, it should be included in the differential diagnosis when patients present with a progressive scrotal mass demonstrating characteristic imaging features of diffusion restriction, heterogeneous hypoechogenicity, and hypervascularity. Notably, despite the well-demarcated contours and regular configuration of early-stage lesions, radical orchiectomy should be strongly recommended over simple epididymectomy given the tumor’s aggressive biological behavior. Simple epididymectomy is associated with higher recurrence rates. Long-term, rigorous surveillance remains paramount for disease management.

## Introduction

Leiomyosarcoma is a malignant neoplasm that can originate from any organ containing smooth muscle tissue. While leiomyosarcoma is relatively common, its occurrence in the epididymis remains exceedingly rare [2, 3]. Kwae et al. [4] reported the first documented case of epididymal leiomyosarcoma in 1949.

The clinical manifestations of epididymal leiomyosarcoma are nonspecific [5]. In its early stages, the lesion may be as small as a soybean, asymptomatic, movable, and easily mistaken for benign conditions. As the tumor enlarges rapidly, it may cause sensations of testicular heaviness and mild discomfort, complicating its differentiation from epididymitis. As the disease progresses, the malignancy intensifies, with tumors beginning to invade adjacent structures. Surgical resection in advanced stages can be particularly challenging, with increased risks of damage to surrounding blood vessels and nerves. In some cases, metastasis to distant sites, such as the extremities and lungs [6], may already be present by this stage, leading to missed opportunities for curative surgery. Therefore, achieving an early and accurate diagnosis is essential for guiding effective treatment.

## Case presentation

A 55-year-old male patient, originally from Syria and residing in China, presented to the Urology Department of the Fourth Affiliated Hospital of Zhejiang University with a left scrotal nodule that had persisted for over a month. The patient reported that approximately one month prior, he had incidentally detected a peanut-sized nodule in his left scrotal area. He noted an absence of significant pain or discomfort in the inguinal or perineal regions.

The patient’s urine color appeared normal. He denied any history of external injury to the inguinal area, as well as any personal or family history of tumors.

On examination, a movable peanut-sized nodule was palpated in the left epididymis, without adhesion, and palpation elicited no significant discomfort. Urinalysis results were normal, as were findings from a complete blood count analysis. Tumor marker testing revealed an elevated carcinoembryonic antigen (CEA) level of 5.2 ng/mL (normal value < 5 ng/mL), AFP was within normal limits.The hCG test was not performed for the patient.

**Fig. 1:**
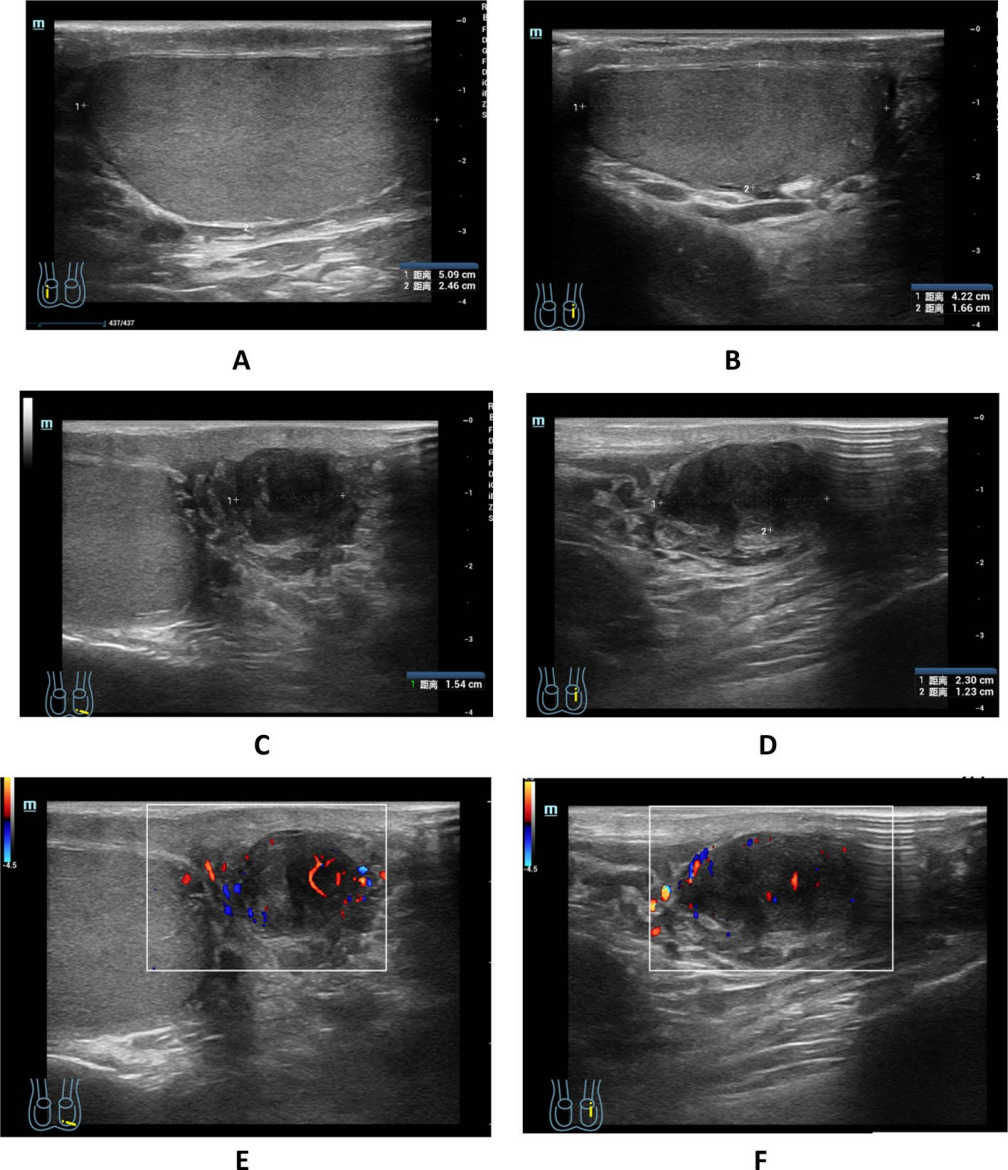
Color Doppler Ultrasonography images of the Epididymal leiomyosarcoma; Normal bilateral testes (**A**, **B**). A low-echoic nodule, approximately 2.30 × 1.23 × 1.54 cm, was identified in the tail of the left epididymis (**C**, **D**), Blood flow signals were detected (**E**, **F**)

**Fig. 2:**
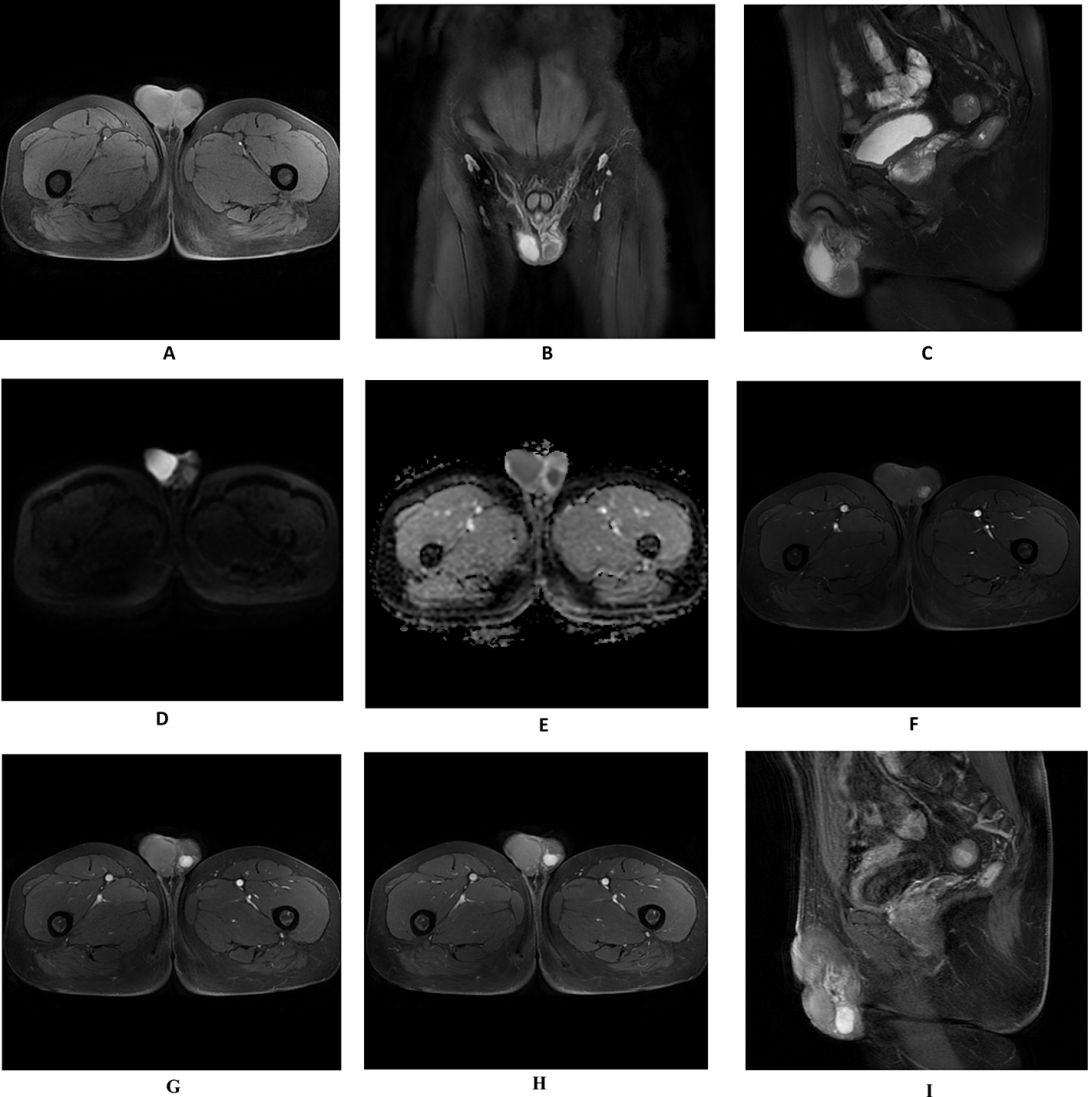
Enhanced Magnetic Resonance Imaging (MRI) scan of the pelvic cavity; Revealed a circular abnormal signal shadow in the left epididymal region, measuring approximately 16 × 12 mm, with well-defined margins. T1WI displayed iso-intense signals (**A**), T2WI showed iso-intense to slightly hyperintense signals (**B**, **C**). DWI exhibited high signal intensity (**D**), ADC map indicated low signal intensity (**E**). Enhanced scan demonstrated progressive enhancement (**F–I**)

In August 2024, the patient underwent Color Doppler Ultrasonography of the scrotum. The right testicle measured approximately 5.09 × 2.46 cm and the left testicle approximately 4.22 × 1.66 cm (Fig. 1A, B). Both testicular capsules appeared smooth with homogeneous internal echotexture, and no significant space-occupying lesions were identified. A low-echoic nodule, measuring approximately 2.30 × 1.23 × 1.54 cm, was visualized in the tail of the left epididymis (Fig. 1C, D), with detectable blood flow signals (Fig. 1E, F). The right epididymis appeared morphologically and dimensionally normal, with clear and symmetrical boundaries, uniform internal echogenicity, and intact structural integrity. A few small anechoic areas were observed within the tunica vaginalis lumen of both testes. And then, the patient underwent an MRI examination, which revealed a circular abnormal signal shadow in the left epididymal area, measuring approximately 16 × 12 mm with well-defined boundaries. The lesion showed iso-intensity on T1-weighted imaging (Fig. 2A), iso-intense to slightly hyperintense signals on T2-weighted imaging (Fig. 2B, C), hyper-intensity on DWI, and hypo-intensity on the ADC map (Fig. 2D, E). Progressive enhancement was observed during the contrast-enhanced scan (Fig. 2F–I).

Following multidisciplinary consultation between the Urology and Radiology departments, the physicians reached a consensus, diagnosing the hypervascularized nodule in the left epididymal region as a neoplastic lesion. The medical team decided to perform a radical orchiectomy for the patient the following day.

The patient underwent left orchiectomy, Under general anesthesia, followed by sterile draping. A 3-cm incision was made in the left scrotum. The skin, subcutaneous tissue, and surface of the tunica vaginalis were carefully dissected in layers, with the tunica being bluntly separated from surrounding tissues. An oval-shaped cystic lesion, approximately 2.5 × 1.5 cm in size, was identified. Complete excision of the testis and epididymis was performed, with satisfactory hemostasis achieved along the lesion margins. The excised specimens were sent for routine pathological examination.

**Fig. 3:**
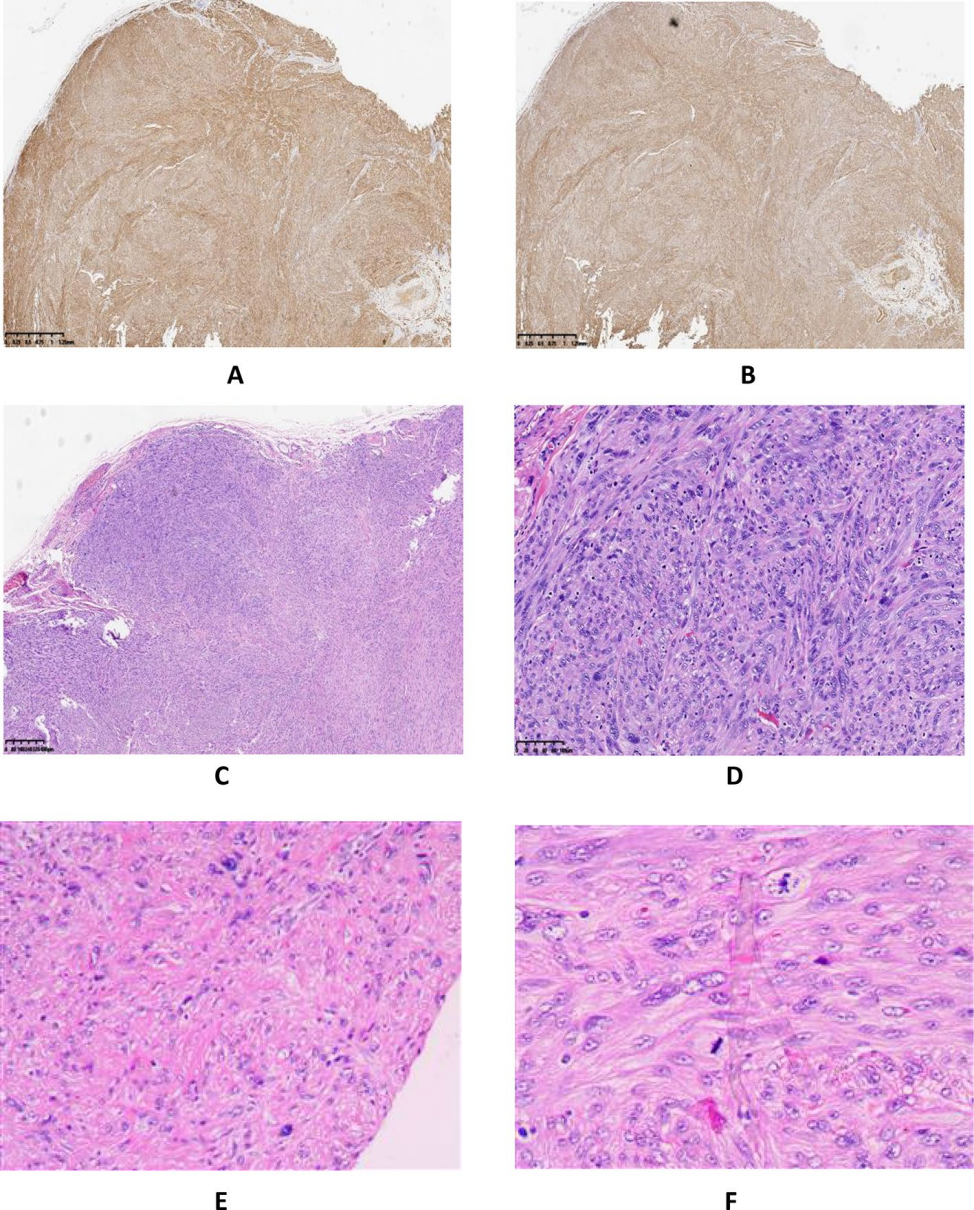
Histopathologic and immunohistochemical of Epididymal leiomyosarcoma; Desmin (positive) (**A**), SMA (positive) (**B**), The pathological diagnosis confirmed an epididymal tumor consistent with a smooth muscle neoplasm, with prominent atypical cells and an increased mitotic index, reaching up to eight mitoses per high-power field. Localized infiltrative growth into the adjacent fibrous tissue was observed, indicative of leiomyosarcoma, with an approximate size of 2.5 × 1.8 cm. The resection margins were negative for malignancy (**C**–**F**)

**Fig. 4:**
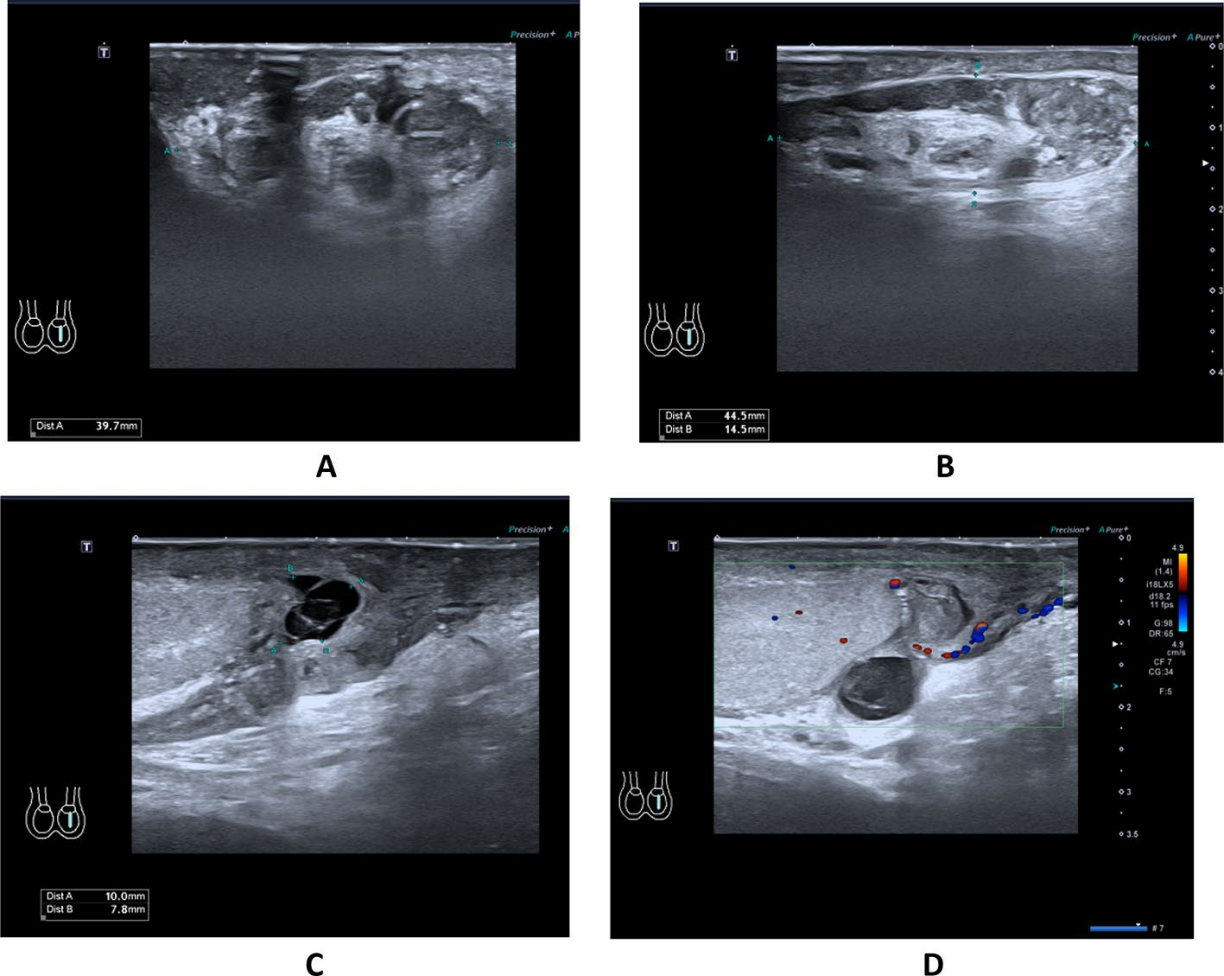
Follow-up ultrasound examination; The surgical site demonstrated disorganized soft tissue structures exhibiting heterogeneous echogenicity, measuring approximately 4.45 × 1.45 × 3.97 cm (**A**, **B**). Multiple anechoic areas were identified, one of which measured approximately 1.0 × 0.78 cm and exhibited poor internal echogenicity with visible septations (**C**, **D**)

**Fig. 5:**
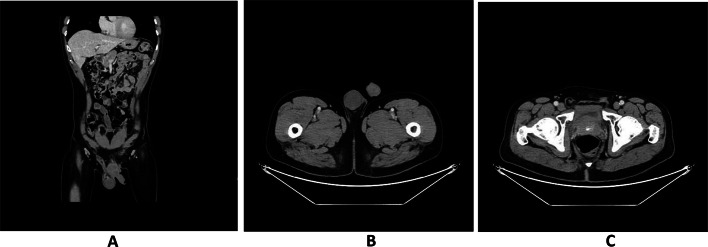
Follow-up CT examination; Absence of left epididymal and testicular (**A**, **B**). Surgical scar at the left inguinal region (**C**)

The postoperative immunohistochemical findings were as follows: Desmin (positive) (Fig. 3A), SMA (positive) (Fig. 3B), Ki-67 (hot spot area at 20% with an average of approximately 10% positive), P53 (wild-type positive), S-100 (negative), CD34 (negative), DOG-1 (negative), CD117 (negative), CK(AE1/AE3) (partially positive), and SDHB (positive). The pathological diagnosis confirmed an epididymal tumor consistent with a smooth muscle neoplasm, characterized by marked atypical cells and an increased number of mitotic figures, up to eight per high-power field, along with localized infiltrative growth into adjacent fibrous tissue, suggesting leiomyosarcoma, with a size of approximately 2.5 × 1.8 cm. Resection margins were negative for malignancy (Fig. 3C–F). During clinical rounds, The patient was instructed to perform regular dressing changes post-discharge (every 2–3 days) and was scheduled for a follow-up appointment in two weeks at the outpatient clinic for monitoring of postoperative recovery. On the scrotal ultrasound follow-up performed in September 2024, postoperative changes consistent with a left radical orchiectomy were observed. The surgical site demonstrated disorganized soft tissue structures exhibiting heterogeneous echogenicity, measuring approximately 4.45 × 1.45 × 3.97 cm (Fig. 4A, B). Multiple anechoic areas were identified, one of which measured approximately 1.0 × 0.78 cm and exhibited poor internal echogenicity with visible septations (Fig. 4C, D). In March 2025, the patient returned for a follow-up examination and underwent a lower abdominal CT scan. The scan revealed surgical scar tissue in the left inguinal region, with clear postoperative structures (Fig. 5C). The left testis and epididymis were absent, and no abnormal enhancement was observed on contrast imaging (Fig. 5A, B). There was no evidence of recurrence or metastatic changes.

## Discussion

Smooth muscle sarcoma, a malignant neoplasm, is relatively uncommon within the urinary system, comprising only 1% to 2% of all malignant tumors in this area [7]. Its occurrence in the epididymis is even rarer, accounting for significantly less than 1%. A review of the literature on PubMed and Sci-Hub over the past decade reveals fewer than 30 reported cases.

Epididymal leiomyosarcoma primarily arises from the smooth muscle tissue within the basement membrane and vascular middle layer of the epididymis, as well as from the smooth muscle tissue of the vas deferens and scrotal wall [8]. These tumors can occur at any age, though they are more commonly observed in individuals aged 60 to 70. Notably, Demetriou et al. [9] reported a case involving a six-year-old boy.

The etiology of epididymal leiomyosarcoma remains unclear. Some researchers propose potential links to anomalies in embryonic development or conditions such as epididymitis, while others suggest associations with prolonged use of high-dose synthetic steroids or previous radiation therapy [10, 11].

According to the literature, the smallest reported tumor its largest diameter only 2 cm [12]. Due to the absence of significant pain, many patients may overlook this condition even if they are aware of the mass. Epididymal leiomyosarcoma exhibits rapid growth and can enlarge significantly within a short time. The largest documented case of epididymal leiomyosarcoma its largest diameter measuring 25 cm associated with an enlarged fistulised inguinal lymph node [13]. As the tumor increases in size, it tends to draw the patient’s attention due to its invasion into nearby blood vessels, nerves, and other structures.

Ultrasonography, owing to its distinct advantages including convenience, real-time dynamic imaging capabilities, and absence of ionizing radiation, has become the most widely utilized modality for diagnosing and differentially diagnosing disorders of the reproductive system. Song et al. [5] reported that epididymal leiomyosarcom demonstrates sonographic characteristics common to many malignant tumors, such as increased density, irregular shape, heterogeneous internal echogenicity, and hypervascularity.It is noteworthy that the lesion in this case, as well as smaller lesions mentioned in some literature, demonstrate regular morphology and well-defined borders. Some lesions even present as well-circumscribed, round or oval-shaped masses, which are indistinguishable from adenomatoid tumors—the most common benign neoplastic lesions of the epididymis.

MR provides improved visualization of the tumor’s size, shape, and boundaries, and offers better insight into the lesion’s internal composition. Mason et al. ’s research suggests that compared to other imaging modalities, MR may demonstrate superior performance in tumor localization and visualizing its structural relationships with surrounding anatomical tissues with greater clarity [14]. Epididymal leiomyosarcoma typically appears as a solid tumor component, showing iso-intense or low signals on T1WI and slightly elevated signals on T2WI. DWI reveals high signal intensity, with low ADC values, indicating restricted diffusion within the tumor mass. Post-contrast imaging demonstrates prominent enhancement patterns with continuous progressive curves. When lesions are small, they often display well-defined borders; however, as they enlarge, they may invade adjacent testicular tissue as well as nearby vascular structures and nerves. Enhanced MRI is particularly valuable for assessing the extent of invasion. MRI also offers significant advantages in detecting metastasis, as metastatic lymph nodes typically show high signals on DWI and heterogeneous enhancement after contrast administration.None of the literature retrieved by the author mentions the MR manifestations of epididymal leiomyosarcoma. This case provides complete and clear MR imaging data, with a small, well-defined lesion. However, it exhibits significant restricted diffusion and marked enhancement. These imaging findings are of great importance for the diagnosis of malignant smooth muscle tumors in the epididymis.

CT enables precise delineation of lesion density characteristics and structural details; it excels in detecting calcifications, particularly microcalcifications, which serve as a key discriminating feature for differentiating epididymal tuberculosis from epididymal leiomyosarcoma [5]. Three-dimensional reconstructed images derived from CT scans comprehensively visualize the lesion and its surrounding anatomical architecture, with superior delineation of vascular distribution patterns that facilitates intraoperative hemorrhage risk mitigation.The definitive diagnosis of epididymal leiomyosarcoma requires pathological examination.The classic histologic features are rhomboid, fasciculate, and braided arrangement of tumor cells, marked cell atypia, and obvious mitosis [15]. Immunohistochemistry: SMA(+), desmin(+), S-100(−), CD34(−), CD117(−) [1].

For confirmed cases of epididymal leiomyosarcoma, prompt surgical intervention is indicated. The standard treatment involves wide radical orchiectomy with high spermatic cord ligation, which maximizes local disease control and minimizes recurrence risk. However, testicular removal inevitably leads to decreased testosterone levels, potentially affecting both sexual function and fertility [16–18]. Notably, Tchienga et al. propose that simple epididymectomy with imaging surveillance represents a viable alternative for tumors exhibiting negative surgical margins, low-grade histology, and localized disease [19].

It is noteworthy that in 2022, Dehghani et al. [20] reported a case with similarities to the present one. Both cases involved the discovery of a painless mass, and no metastatic signs were detected on auxiliary examinations, followed by surgical intervention. The difference lies in the fact that the patient in the reported case initially underwent scrotal surgery for specimen excision, with pathological results confirming epididymal leiomyosarcoma, leading to a second procedure (radical inguinal orchiectomy with high ligation of the spermatic cord). This approach notably increases the risk of tumor seeding and metastasis, in addition to causing psychological trauma to the patient.

Adjuvant therapies encompass radiotherapy, chemotherapy, and targeted therapy. Radiotherapy is indicated for postoperative patients with high-risk factors (such as positive margins) to reduce local recurrence risk. While this approach has demonstrated efficacy in limb soft tissue sarcomas, its effectiveness in epididymal leiomyosarcoma lacks validation through large-scale studies. In the literature reviewed by the authors, some early-stage cases did not receive radiotherapy and showed no signs of recurrence or metastasis, whereas some advanced cases experienced recurrence or metastasis within 1–3 years despite undergoing radiotherapy. Furthermore, Jonker-Pool et al.’s research suggests that.

radiotherapies are significantly associated with adverse effects on libido, orgasmic intensity, erectile sustainability, and semen volume [18]. Chemotherapy is primarily utilized for advanced-stage patients to control systemic metastases. Targeted therapy, which focuses on specific genes or pathways, represents a frontier in research. Clinical trials are currently investigating the efficacy of targeted agents such as XL092 (Zanzalintinib) in advanced leiomyosarcoma, and we will continue to monitor the progress of this research.Long-term, regular surveillance is strongly recommended across all available literature due to the documented risk of delayed recurrence and metastasis.

## Conclusion

Epididymal leiomyosarcoma presents significant diagnostic challenges in clinical practice due to the non-specificity of its clinical manifestations and imaging features. When a patient exhibits a progressively enlarging mass in the epididymal region with imaging characteristics including diffusion restriction, heterogeneous hypoechogenicity, and hypervascularity, this entity should be considered in the differential diagnosis. Upon confirmation of diagnosis, surgical intervention is imperative. Considering the highly aggressive nature of the disease, radical orchiectomy was performed.Critically, long-term and regular surveillance cannot be overemphasized.

## Data Availability

The original contributions presented in the study are included in the article/supplementary material. Further inquiries can be directed to the corresponding authors.
